# A program for audit of infections and antibiotic resistance in critical ill

**DOI:** 10.1186/1753-6561-5-S6-O6

**Published:** 2011-06-29

**Authors:** HS-I Hanberger, G Fransson, S Walther

**Affiliations:** 1 Infectious Diseases, Clinical experimental medicine, Linköping, Sweden

## Introduction / objectives

Infections caused by antibiotic resistant pathogens is a growing concern that complicates treatment and influences patient outcome in critical ill. The purpose of this work was to develop and test the feasibility of a system for continuous audit of resistance and compliance to antibiotic treatment guidelines for severe infections in critical care.

## Methods

Records from ICU patients (including i.e. reason of admission, illness severity score, interventions and nursing workload, adverse events, outcome in the ICU) were collected in the Swedish Intensive Care Registry (SIR). Mortality data was added weekly for each episode of care (EOC). A web service was developed for individual collection of results from microbiological cultures and analyses for each EOC. Development of the web service included a detailed and cumbersome translation of laboratory specific nomenclatures for samples, cultures, microbials and methods.

## Results

Bacteriological data from clinical microbiological laboratories were merged with its respective EOC in SIR yielding data on patient-specific bacteriology and microbial susceptibility. Trends in microbial resistance among alert pathogens were examined. Data on gender, age, co-morbidity were collected from SIR and correlated to microbiological findings and 30-day mortality. Resistance patterns were analyzed to assess the appropriateness of National guidelines for the treatment of pneumonia in the ICU showing 86 % possible accuracy with suggested treatment for community acquired and 82 % for hospital acquired disease.

## Conclusion

Microbial findings matched with reason of ICU admission can be used to validate or update national guidelines for proper antibiotic treatment. This program may be developed to an early warning system for antibiotic resistance in intensive care

## Disclosure of interest

None declared.

